# What is the optimal outcome after endoscopic sinus surgery in the treatment of chronic rhinosinusitis? A consultation of Canadian experts

**DOI:** 10.1186/s40463-021-00519-9

**Published:** 2021-06-16

**Authors:** Nadim Saydy, Sami P. Moubayed, Marie Bussières, Arif Janjua, Shaun Kilty, François Lavigne, Eric Monteiro, Smriti Nayan, Marilou Piché, Kristine Smith, Doron Sommer, Leigh Sowerby, Marc A. Tewfik, Ian J. Witterick, Erin Wright, Martin Desrosiers

**Affiliations:** 1 Division of Otolaryngology – Head & Neck Surgery, Centre Hospitalier de l’Université de Montréal, Université de Montréal, Montreal, Quebec Canada; 2 Division of Otolaryngology – Head & Neck Surgery, Maisonneuve-Rosemont Hospital, Université de Montréal, Montreal, Quebec Canada; 3 Division of Otolaryngology – Head & Neck Surgery, Centre Hospitalier de l’Université de Sherbrooke – Hôtel-Dieu Hospital, University of Sherbrooke, Quebec, Canada; 4 Division of Otolaryngology – Head & Neck Surgery, Vancouver General Hospital, University of British Columbia, Vancouver, British Columbia Canada; 5 Division of Otolaryngology – Head & Neck Surgery, The Ottawa Hospital, University of Ottawa, Ottawa, Ontario Canada; 6 Division of Otolaryngology – Head & Neck Surgery, Mount Sinai Hospital, University of Toronto, Toronto, Ontario Canada; 7 Division of Otolaryngology – Head & Neck Surgery, Cambridge Memorial Hospital, McMaster University, Hamilton, Ontario Canada; 8 Division of Otolaryngology – Head & Neck Surgery, Hôpital Saint-Sacrement, Laval University, Quebec, Quebec Canada; 9 Division of Otolaryngology – Head & Neck Surgery, Winnipeg Health Sciences Center, University of Manitoba, Winnipeg, Manitoba Canada; 10 Division of Otolaryngology – Head & Neck Surgery, St-Joseph’s Hospital, Western University, London, Ontario Canada; 11 Division of Otolaryngology – Head & Neck Surgery, Jewish General Hospital, McGill University, Montreal, Quebec Canada; 12 Division of Otolaryngology – Head & Neck Surgery, Walter C MacKenzie Health Sciences Center, University of Alberta, Edmonton, Alberta Canada

**Keywords:** Chronic rhinosinusitis, Endoscopic sinus surgery, Patient-centered care, Short title: Impressions of endoscopic sinus surgery.

## Abstract

**Objectives:**

Many experts feel that in the absence of well-defined goals for success, they have an easier time identifying failure. As success ought to not be defined only by absence of failure, we aimed to define optimal outcomes for endoscopic sinus surgery (ESS) in chronic rhinosinusitis (CRS) by obtaining expert surgeon perspectives.

**Methods:**

A total of 12 surgeons participated in this targeted consultation. Face to face semi-structured interviews were performed with expert surgeons in the field of CRS and ESS. General impressions and personal definitions of acceptable operative success and optimal operative outcomes were compiled and summarized.

**Results:**

According to an expert survey, patients’ main objectives are an improvement in their chief complain, a general improvement in quality of life (QoL), and a better overall symptomatic control. The most important aspects of endoscopy for defining a successful intervention were an adequate mucus circulation, a healthy mucosa, minimal edema, and patency of all explored cavities or ostia. In the assessment of surgical outcomes, it was determined that both objective and patient reported data must be carefully examined, with more attention given to subjective outcomes.

**Conclusions:**

According to data gathered from a Canadian expert consultation, a definition of success must be based on both subjective data and nasal endoscopy. We propose to define an acceptable outcome as either a subjective improvement of at least the minimal clinically improvement difference of a validated patient reported outcome questionnaire, along with a satisfactory endoscopic result (1) or a complete subjective resolution with a sub-optimal endoscopy (2).

**Graphical abstract:**

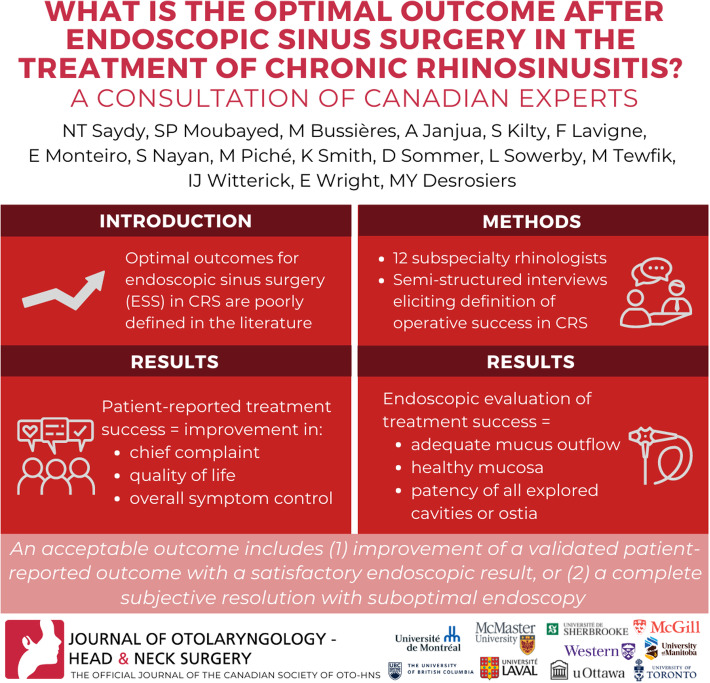

## Background

Chronic rhinosinusitis (CRS) is an inflammatory condition of the nasal and paranasal mucosa with a prevalence of 5% in Canada, and up to 10–12% in the United Kingdom and the United States [1–3]. Despite low mortality rates, its impact on patients’ quality of life (QoL) and health related quality of life (HRQoL) is considerable [4]. Common symptoms include facial congestion, purulent discharge, facial pain, nasal obstruction and headaches [5]. Associations between CRS and depression, anxiety, olfactory dysfunction, fatigue, sleep disturbance and sexual dysfunction have also been reported [6–10]. Moreover, CRS is associated with a significant economic burden. In 2014, healthcare related costs for CRS in the US were between 6.9 and 9.9 billion US$. Indirect costs, namely absenteeism and loss of productivity, were estimated at 13 billion US$ [11]. In Canada, the total cost related to CRS is close to 2 billion CAN$ [12].

One of the cornerstones of the treatment of CRS is control of inflammation. Both medical and surgical interventions aim to restore a healthy mucosa and adequate drainage pathways for secretions. Since its introduction in 1985, endoscopic sinus surgery (ESS) has quickly become the mainstay of surgical treatment for CRS [13, 14], with between 10,000 and 15,000 Canadian patients undergoing ESS annually [15]. Extensive research shows that ESS in selected patients is associated with an improvement in CRS symptoms, QoL, sleep and even cognitive dysfunction [16–20]. Due to the very broad range of clinical presentations, many key concepts in the diagnosis, management and outcome measures of CRS remain without widely accepted definitions. One of these is the definition of an acute exacerbation in CRS. While experienced clinicians tend to recognize it when they see it, the lack of a clear definition is an obstacle to research and the improvement of directed therapies [21]. The same is true of the definition of success following ESS; we recognize it when we see it, but it is difficult to state clearly.

Although many studies show mean improvements in patient-reported outcome measure (PROM) questionnaires postoperatively, some patients still report a lack of improvement after surgery or even a worsening in symptoms. With a recent paradigm shift towards a patient-centered approach to clinical decision making, research groups have begun studying the patient-experience of CRS. In a qualitative study of patient views and experiences of current management of CRS in the United Kingdom*,* side-effects and limitations of surgery was raised by patients as an important theme [22]. With the development of novel therapies and the improvement of surgical technique, new benchmarks for success must be defined. Both the *2020 EPOS* guidelines and the *Quality Improvement Committee of the American Rhinologic Society* have suggested that more work is required to develop measures of quality for CRS and further characterize its impact [23, 24]. An understanding of both patient experience and surgeon experience is necessary to construct clinically relevant tools to measure and define surgical success. The *Choosing Wisely Canada* campaign is a great initiative from a group of national leaders within the subspecialty, which aims at reducing unnecessary diagnostic testing and therapeutic interventions in rhinology. These consensus recommendations are an important step in standardizing the management of acute rhinosinusitis (ARS) and nasal fractures. We believe that standardization is important not only in the diagnosis and treatment of complex diseases like ARS and CRS, but also in the measure of treatment outcomes [25].

There is no widely accepted definition of what constitutes an optimal outcome after ESS. We believe that outcome measures will eventual help us determine which patients would benefit from which treatment. In order to measure outcome measures, there needs to be clear definitions of an optimal outcome, and acceptable outcome. In this study, we aimed to explore expert surgeons’ perspectives on various themes pertaining to successful ESS in patients with CRS. We restricted this targeted consultation to expert surgeons. Patient impressions will be collected separately to achieve a comprehensive understanding of success and optimal outcomes. Ultimately, we sought to propose a “surgeon definition” of the optimal outcome, as well as what constitutes an acceptable outcome.

## Methods

### Design

This study was reviewed and approved by the University of Montreal Health Center (CHUM) Institutional Review Board. A total of 12 surgeons participated in a targeted consultation of experts. They were contacted by email prior to the 72nd Annual CSO-HNS (*Canadian Society of Otolaryngology – Head & Neck Surgery*) Meeting, in 2018. Expert surgeons in the field of CRS and ESS present at the conference and willing to participate were met during face-to-face semi-structured interviews, scheduled to last approximately 20 min each. Expert surgeons were all Otolaryngologists – Head & Neck Surgeons with a specialized practice in Rhinology and Skull Base Surgery in Canada. All interviewed surgeons are fellowship-trained professors in tertiary care practice within academic centers. Interviews were conducted either in English or French depending on surgeon preference. An emphasis was placed on individual criteria used by experts to define operative success. No financial compensation was offered. Written consent was obtained from all participants. Interviews were audio-recorded and transcribed word-for-word; field notes were taken during interviews.

### Interview templates

Based on a literature review, the interviewer (NS) produced a template for interviews (Appendix). Each question was evaluated by the senior author (MD); items were subtracted, and wording was modified to ensure that common concerns raised by patients during clinical visits were addressed. The template of the interview was composed of themes we wished to explore in order to generate meaningful discussion. It was based on a review of the literature of outcome measures in CRS and in other surgical pathologies, patient-reported outcome measures in CRS, and other criteria used in CRS research to define success or a positive surgical outcome in ESS.

An iterative process was conducted in concert by NS and MD throughout the interviews to remove low-yield questions or add prompts. Issues identified in early interviews were corrected for subsequent interviews. Whenever experts did not understand the question, the interviewer provided clarification or reformulated the question. In order to explore the various themes spontaneously raised by experts, initial questions were open-ended. Specific prompts were added to stimulate discussion and provide a structure. Moreover, this allowed experts to provide impressions on themes and topics they had not spontaneously mentioned.

## Results

Of the 12 surveyed participants, 8 (67%) were male. The interview was performed in French with 3 (25%) surgeons. Duration of interviews was between 10 and 29 min. Experts first described what they believe are patients’ main goals and expectations. All the goals of patients according to experts are listed in Table 1. The most cited goals are an improvement in patients’ chief complain (50%), improvement in QoL and HRQoL (42%), and a better overall symptomatic control (33%). They reported patients also hope to decrease the number and severity of acute exacerbations (25%) and decrease the need for topical and rescue medication (17%). Three experts (25%) spontaneously mention that goals for patients with CRSwNP and CRSsNP tend to be different.

**Table 1:** Physician-perceived Objectives of Patients Undergoing Endoscopic Sinus Surgery Ranked by Number of Times Mentioned by Interviewed Experts

Patient Goals	Number of times mentioned (%)
Improving in the most bothersome symptom	6 (50%)
Improving general quality of life	5 (42%)
Having an overall improvement in symptoms	4 (33%)
Minimizing the number and/or severity of acute exacerbations	3 (25%)
Improving quality of sleep	2 (17%)
Requiring less/no medical treatment	2 (17%)
Decreasing absenteeism	1 (9%)
Forgetting about their disease	1 (9%)
Having no rhinologic symptoms	1 (9%)
Having a better delivery of topical medication	1 (9%)
Being more productive	1 (9%)
Getting rid of the chronic malaise	1 (9%)
Reaching a level of inflammatory control that they cannot achieve with only medication	1 (9%)

All 12 interviewed experts (100%) believe that a definition of operative success must be based on both subjective patient-reported measures and objective physician-recorded measures. All 12 experts (100%) routinely use nasal endoscopy to assess surgical outcomes. There is a high variability in the scales used by experts to grade post-operative endoscopy. Some experts use a personal grading system; others use a validated endoscopic score, like the Lund Kennedy Endoscopic Score (LKES), the Modified Lund Kennedy (MLK) or Perioperative Sinus Endoscopy (POSE). Some experts also use part of validated score, for example the edema rating portion of the MLK. Most experts stated that a numerical value does not help them routinely in the clinical decision making or in their personal assessment of success. Almost all experts seldom use these scales in a clinical setting and favor the use of an individual grading or a description of the post-operative cavities in their own words. According to experts, the most important criteria for a satisfactory endoscopic result are an adequate mucus drainage, a healthy mucosa without edema, patent ostia with open cavities which can be visualized because of an adequately medialized middle turbinate. Patients should ideally not have any evidence of purulence, or recurrence or persistence of polyps. All criteria to define a perfect endoscopic result are listed in Table 2. No other objective measure is used routinely by any of the experts in the clinical setting. For all experts, radiological modalities, such as CT-imagining, should only be considered for suspected complications or patients whose symptoms worsened following surgery.

**Table 2:** Criteria for an Optimal Endoscopic Result Following Endoscopic Sinus Surgery

Criteria	Number of times mentioned (%)
Adequate mucus drainage with lack of synechiae, scarring and mucus recirculation	11 (92%)
Healthy mucosa and lack of edema	8 (67%)
Patency of all explored cavities and ostia	7 (58%)
Adequate middle turbinate position with no obstruction of visualization	6 (50%)
Absence of polyps in the olfactory cleft	5 (42%)
Lack of purulence	5 (42%)
Reduction in turbinate size	2 (17%)
Well defined cavities/partitions	2 (17%)
Lack of crusting	1 (9%)
Visualization into the frontal sinuses	1 (9%)
Crisp ethmoidal partitions	1 (9%)
Visualization into the maxillary antrostomies	1 (9%)

Experts tend to collect subjective data in a non-standardized fashion during medical history, with an emphasis on preoperative complaints. Rhinologic symptoms are systematically questioned by all experts: congestion, pressure, obstruction, and nasal discharge. Other important symptoms that some experts systematically question are olfaction, quality of sleep, functionality, mood and energy level. Any other patient complaints are discussed and characterized. Many experts expressed an interest in eventually implementing routine use of patient-reported outcome measures (PROM) in the clinical setting. Thus far, 1 expert (9%) uses the Sinonasal Outcome Test (SNOT-22) systematically at all visits with CRS patients. The SNOT-22 is a validated CRS-specific PROM which contains disease-specific and general quality of life items [26]. Reported limitations to the implementation of PROM include length of administration, patient burden, administrative issues and inherent complexity of existing PROM. No other validated PROM questionnaire is routinely used in the clinical setting by the experts.

No expert uses an algorithm or formula to weigh surgical success according to subjective and objective data. That said, all experts believe that subjective data should carry more weight in defining success than objective data. According to experts, early post-operative success is principally determined by complete endoscopic clearance of disease and avoidance of operative complications. Long term success for ESS is more difficult to define but some of the items proposed are inflammatory control with medical therapy, long-term symptomatic relief, avoidance of reoperation, and diminished number of flare-ups. All experts agree that the ideal surgical outcome could be defined as an optimal post-operative nasal endoscopy, combined with a complete symptomatic resolution. Likewise, all experts agree that a poor endoscopic result with identical or worsened symptoms constitutes a surgical failure. Most experts believe that a good endoscopic result without an improvement in symptoms does not constitute an acceptable outcome. In cases where patient symptoms are significantly improved, but endoscopy is poor or sub-optimal, opinions vary more widely. Some experts believe that patient satisfaction is by far the most important criterion and that a satisfied patient translates to a successful surgery. Others argue that we cannot claim the surgery was successful if there are objective signs of failure or impending failure on endoscopy, even in the absence of subjective concerns.

## Discussion

No clear minimal number of interviews exists in qualitative research to achieve thematic saturation. That said, many agree that 12 interviews are sufficient if the participants are homogeneous [27]. Given the homogeneous nature of the expert group, interviews were conducted with 12 experts. In the field of oncologic surgery, arguably the most important measure of the success of an intervention is disease-free survival, or relapse-free survival. In the field of cardiac surgery, patients are most at risk immediately post-operatively; thus, 30-day mortality is used as an important benchmark for the success of an intervention. In our field, CRS is associated with a low risk of mortality and routine ESS is a safe surgery, with approximately 0.5–1% risk of major complications [28, 29]. We need to determine other criteria for success other than rates of morbidity and mortality, since ESS is a quality of life intervention that should not be performed in patients with significant per-operative risk.

This work is analogous to the research currently being performed in the field of obstructive sleep apnea (OSA). In OSA, it is now recognized that multiple other criteria beside apnea-hypopnea index must be considered when defining success in OSA surgery [30]. Pang & Rotenberg even proposed a comprehensive set of success parameters with the acronym *SLEEP GOALS* which looks at other factors like oxygenation, blood pressure and the Epworth sleepiness scale [31]. This dichotomy between objective measures and validated patient questionnaires is not unique in the field of Otolaryngology – Head & Neck Surgery to outcome measures. In attempting to define acute acerbations in CRS, Wu et al. examined SNOT-22 scores as well as many objective outcomes, including expression of mucus cytokines and eosinophil count [21],

In our study, we identified the two modalities that experts believe should dictate our appreciation of surgical success: nasal endoscopy and patient-reported outcomes. The lack of association between these two components has been the topic of extensive research. While DeConde et al. have shown that certain sub-domains of the SNOT-22 can be correlated to LKES scores using canonical analysis [32], a large body of evidence shows little or no association between patient symptoms and endoscopic evaluation using different PROM and endoscopic scoring systems [33–35]. In the face of often contradicting information, it is can be difficult to set priorities, or even to determine if an intervention was successful. While there is no solid evidence to support these claims, many experts stated that in their experience endoscopic changes predict a deterioration of symptoms in patients. Thus, for an identical level of postoperative symptomatic improvement, a patient with a better endoscopy would likely have a better chance of long-term disease control with continued use topical medication. That said, there are pitfalls to relying too heavily on objective outcomes like endoscopy in a disease like CRS. Many patients have comorbid diagnoses of depression and experience chronic pain [6, 36]. Evidence shows these patients have significantly lower gains with regards to sino-nasal quality of life following ESS. This further complicates the evaluation of postoperative outcomes, since they seem to be partly tied to preoperative characteristics.

Another interesting concept mentioned spontaneously by some experts is the notion of a *moving target*. While patients might initially complain of one specific problem, they may present postoperatively dissatisfied with a completely different problem. Whether the new problem arises as a result of surgical intervention or whether it was present, but simply not noticed is not clear. In any case, many patients are perpetually dissatisfied because their goals change after each surgical intervention. This further emphasizes the crucial role of pre-operative counselling, and the relationship between success and patient goals. Indeed, experts believe that patients’ most important objective is usually an improvement in one very bothersome symptom. A critic against solely relying on PROM numerical values to define the success of an intervention is the fact that all items are weighed identically, when in fact their relative importance varies from patient to patient. Indeed, experts explain that in their experience, patients with CRS with nasal polyposis (CRSwNP) often seek an improvement in breathing and olfaction while those with CRS without nasal polyposis (CRSsNP) tend to experience more facial pain and discharge.

With the rise of biologic agents and the improvements in surgical techniques, it is very likely that outcomes will vastly improve in the near future. Initiatives to improve selection of surgical candidates and the definition of quality indicators in CRS are also in important step in the patient-centered approach to future care. Cottrell et al. used a guidelines-based approach to define 9 quality indicators for CRS, which will support improvements in quality of care and accountability [37]; Mattos et al. offer a framework for an appropriate pre-surgical algorithm, which will help offer this effective therapy to the right patients [38]. It is important that we have tools that allow us to set clear goals for the future. With tools that allow us to define the optimal outcome, we may shift the view of CRS as an incurable disease that may be improved temporarily with surgery to that of a disease for which complete control may be obtained with optimal surgical and medical therapy.

Because success in ESS is much harder to conceptualize than failure, a lot of research has thus far focused around identifying common traits in patients who do not respond to surgical therapy. This has led the identification of numerous disease endotypes. Studies show that there are different clinical and pathologic features to these different endotypes. It is more and more apparent that these different endotypes differ in terms of treatments and prognosis. That said, the distinction is rarely made clinically, partly because of a lack of strategies for differentiation [39]. This most likely partially explains the important and often unpredictable differences in post-operative outcomes amongst various patients. This very complex domain may have discouraged clinicians from attempting to personalize treatment for CRS patients. That said, we know there are patients whom for some reason respond perfectly to therapy. We sought to define what the optimal outcome is, to eventually identify which patients are “very-good responders” to surgical treatment and add to the growing body of work in the field of quality of care research in CRS.

Patient-reported outcome measures are validated questionnaires which are performed preoperatively and postoperatively. The difference in scores corresponds to the improvement in symptoms experienced by the patient. The minimal clinically important difference (MCID) corresponds to the minimal difference a patient is able to perceive. For the SNOT-22, the MCID is 12 in medically managed CRS patients and 9 in patients following ESS [26, 40]. Mattos et al. showed that in a cohort of 100 CRS patients undergoing ESS, postoperative satisfaction as defined by a willingness to undergo ESS again did not correlate with MCID. The most important symptoms in these patients were rhinogenic, smell and sleep related symptoms [41]. This shows that patient satisfaction is a complex concept that may not be directly correlated to symptom severity.

**Fig. 1:**
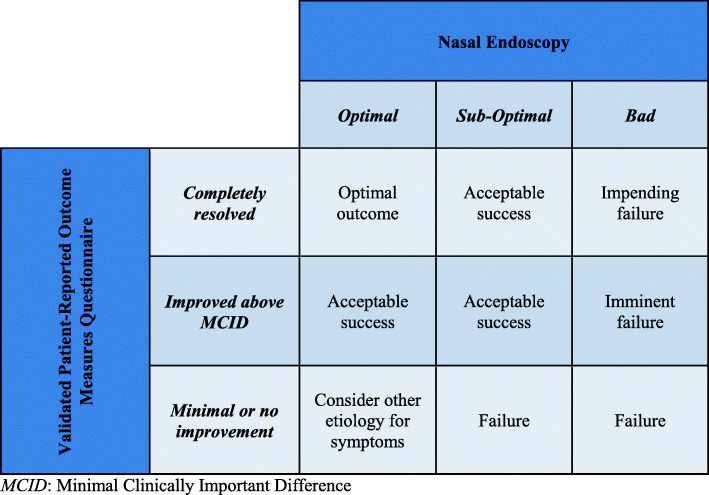
Defining Post-Operative Success and Failure in Endoscopic Sinus Surgery. *MCID*: Minimal Clinically Important Difference

**Fig. 2:**
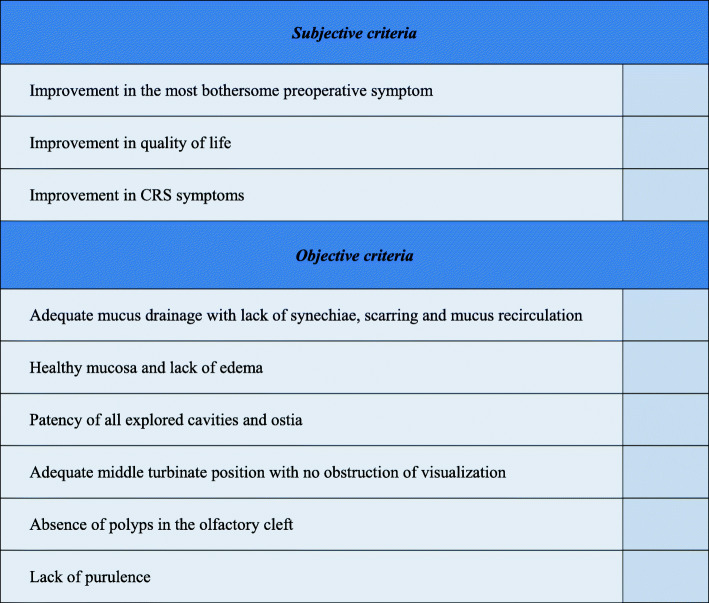
Checklist for the assessment of Post-Operative success following Endoscopic Sinus Surgery

We propose definitions for an optimal outcome and acceptable success after ESS in Fig. 1. We also propose a checklist, which we suggest could be used as a reminder for clinicians to specifically evaluate post-operative outcomes. It can also serve as a guide for documentation of the important aspects of post-operative success (Fig. 2). The checklist includes subjective and objective elements which were brought up by experts (at least 25%) as being important factors for the attainment of an optimal post-operative outcome after ESS. A more detailed definition or the creation of a precise clinical tool were impossible considering this study’s limitations. The first and most important limitation is inherent to the qualitative nature of the study. That said, this semi-structured interview format provided the opportunity to explore multiple themes and to allow experts to share their experience in their own words. Thus, there was a hypothesis generating aspect to this study. Another limitation is that we still do not a have a formal definition of *optimal*, *sub-optimal* and *bad* outcomes for nasal endoscopy. Furthermore, we do not know the ideal timeframe when we should try to define success and suggest that this ought to be on ongoing process during clinical follow-up, though most experts claimed it should be within 3 to 6 months. Finally, we do not have a consensus on which if a validated PROM should be used, and which one. Despite these limitations, this work allowed us to provide foundational elements that we believe must be taken into account in further studies aiming to provide a validated tool to quantitatively measure success.

Though further investigation is warranted, we believe the findings of this study add some interesting points to outcomes research in CRS. We have found that a composite score – composed of a nasal endoscopic score and preoperative and postoperative PROM questionnaire scores – is necessary if we eventually aim to accurately quantify success after ESS. In this composite score, subjective data should be weighed more heavily than objective data. To conclude, patients’ perspective should be taken into account in the development and validation of this tool.

## Data Availability

Data used in the current study is available from the corresponding author on reasonable request.

## Appendix

### Expert interview template

1. Please provide your definition of “operative success” after ESS.
2. Please provide your definition of a “perfect operative result” after ESS.
3. Please describe what objective modalities you use to assess success after ESS for patients with CRS.
  Prompt: Please provide a definition of operative success according to each of these modalities.
  Prompt: Please provide a definition of a perfect operative result according to each of these modalities
4. Please describe what subjective data you collect to assess success after ESS for patients with CRS.
  Prompt: Do you use patient-reported outcome measures?
  Prompt: If so, please explain the strenghts and weaknesses for each questionnaire.
5. Please explain how you weigh each element of operative success after ESS in your decisional algorithm.
  Prompt: How important is your “gut-feeling” in determining success?
6. What aspects of ESS do you believe matter most to patients?
7. Please describe the 5 best questions to determine success after ESS according to patients’ symptoms and experience of disease
8. Please explain how you assess operative success using nasal endoscopy.
  Prompt: Do you use a grading system?
  Prompt: If so, please explain the strenghts and weaknesses for this grading system.
  Prompt: If so, please explain why you do not use other grading systems.
9. Please describe a “perfect endoscopic result” after ESS.
10. Please list the best questions to assess patients’ nasal symptoms after ESS.
  Prompt: How would you rank them in order of importance?
11. Please list the best questions to assess patients’ change in sleep after ESS.
  Prompt: How would you rank them in order of importance?
12. Please list the best questions to assess patients’ change in olfaction after ESS.
  Prompt: How would you rank them in order of importance?
13. Please list the best questions to assess patients’ pain after ESS.
  Prompt: How would you rank them in order of importance?
14. Please list what you believe are the most important aspects of ESS according to patients.
  Prompt: How would you rank them in order of importance?
15. Please describe the goals of patients undergoing ESS.
16. Please describe the expectations of patients undergoing ESS.
17. Please describe what you believe is patients’ definition of operative success after ESS.
18. Do you have a standardized follow-up for patients who underwent ESS?
19. What would be the ideal recall time when questionning patients about their symptoms?

## Abbreviations

ARS: Acute rhinosinusitis
CRS: Chronic rhinosinusitis
CRSsNP: Chronic rhinosinusitis without nasal polyps
CRSwNP: Chronic rhinosinusitis with nasal polyps
ESS: Endoscopic sinus surgery
HRQoL: Health-related quality of life
LKES: Lund-Kennedy endoscopic score
MCID: Minimal clinically important difference
MLK: Modified Lund-Kennedy
POSE: Perioperative sinus endoscopy
PROM: Patient-reported outcome measure
QoL: Quality of life
SNOT-22: Sinonasal outcome test
